# *De novo* assembly and analysis of the transcriptome of *Ocimum americanum* var*. pilosum* under cold stress

**DOI:** 10.1186/s12864-016-2507-7

**Published:** 2016-03-09

**Authors:** Xiangqiang Zhan, Lan Yang, Dong Wang, Jian Kang Zhu, Zhaobo Lang

**Affiliations:** State Key Laboratory of Crop Stress Biology for Arid Areas and College of Horticulture, Northwest A&F University, Yangling, Shaanxi 712100 China; Shanghai Center for Plant Stress Biology, Shanghai Institutes for Biological Sciences, Chinese Academy of Sciences, Shanghai, 200032 China; Department of Horticulture and Landscape Architecture, Purdue University, West Lafayette, IN 47907 USA

**Keywords:** Chilling tolerance, RNA-seq, Gene regulation, *O. americanum*

## Abstract

**Background:**

*Ocimum americanum* var*. pilosum* is a chilling-sensitive, widely distributed plant that is consumed as a vegetable in central and southern China. To increase our understanding of cold stress responses in this species, we performed *de novo* transcriptome assembly for *O. americanum* var*. pilosum* and compared the transcriptomes of plants grown under normal and low temperatures.

**Results:**

A total of 115,022,842 high quality, clean reads were obtained from four libraries (two replicates of control samples and two replicates of chilling-treated samples) and were used to perform *de novo* transcriptome assembly. After isoforms were considered, 42,816 unigenes were generated, 30,748 of which were similar to known proteins as determined by a BLASTx search (E-value < =1.0E-05) against NCBI non-redundant, Swiss-Prot, Gene Ontology, KEGG, and Cluster of COG databases. Comparative analysis of transcriptomes revealed that 5179 unigenes were differentially expressed (with at least 2-fold changes, FDR < 0.01) in chilling-treated samples, and that 2344 and 2835 unigenes were up- and down-regulated by chilling stress, respectively. Expression of the 10 most up-regulated and the five most down-regulated unigenes was validated by qRT-PCR. To increase our understanding of these differentially expressed unigenes, we performed Gene ontology and KEGG pathway enrichment analyses. The CBF-mediated transcriptional cascade, a well-known cold tolerance pathway, was reconstructed using our *de novo* assembled transcriptome.

**Conclusion:**

Our study has generated a genome-wide transcript profile of *O. americanum* var*. pilosum* and a *de novo* assembled transcriptome, which can be used to characterize genes related to diverse biological processes. This is the first study to assess the cold-responsive transcriptome in an *Ocimum* species. Our results suggest that cold temperature significantly affects genes related to protein translation and cellular metabolism in this chilling sensitive species. Although most of the CBF pathway genes have orthologs in *O. americanum* var*. pilosum,* none of the identified cold responsive (COR) gene orthologs was induced by cold, which is consistent with the lack of cold tolerance in this plant.

**Electronic supplementary material:**

The online version of this article (doi:10.1186/s12864-016-2507-7) contains supplementary material, which is available to authorized users.

## Background

Species in the genus *Ocimum* are native to Africa, South America, and Asia, and are valued as aromatic and medicinal plants. Essential oils extracted from *Ocimum* spp., such as *O. basilicum*, *O. sanctum*, and *O. americanum*, have anti-inflammatory, antimicrobial, antioxidant, and larvicidal activities [1–4]. Recently, the transcriptomes of *O. basilicum* and *O. sanctum* were assembled and used to identify genes involved in the biosynthesis of essential oils and medicinal metabolites [5, 6]. In addition to being used for medicinal purposes in some parts of the world [2–4], *O. americanum* var*. pilosum* of the Lamiaceae family is one of the most popular vegetables in Anhui and Henan provinces in China, where it is frequently grown. *O. americanum* var*. pilosum* is very sensitive to chilling injury, however, which limits its growing area and can also dramatically reduce its yield, leading to economic losses for farmers.

Low temperature is a major environmental factor determining the growth and productivity of plants. Temperate plants are tolerant to chilling temperatures (0-15 °C) but are usually intolerant of freezing temperatures (<0 °C) [7]. For many temperate plant species, a period of exposure to chilling temperatures increases plant tolerance to subsequent freezing conditions in a phenomenon known as “cold acclimation” [7]. In contrast, plants of tropical and subtropical origins are intolerant of chilling and freezing temperatures. In response to low temperature, many biochemical and physiological processes change in plants through regulation of cold responsive (COR) gene expression as well as through posttranslational protein modifications. The ICE1-CBF-COR transcriptional cascade (inducer of CBF expression 1 and C-repeat binding factor transcriptional cascade) is the best characterized pathway for gene regulation under cold conditions in many species [8]. In *Arabidopsis*, three transcription factors in the CBF family (*CBF1*, *CBF2*, and *CBF3*) are rapidly induced by low temperatures [9]. These CBFs can bind to and activate downstream *COR* genes, such as *COR15*, *COR47*, *COR78*, and *KIN1*, to protect plant cells from freezing damage. The pathway may also be important for chilling tolerance [10]. Under cold conditions, the expression of *CBFs* can be regulated by several upstream transcription factors such as ICE1, CAMTAs, MYB15 and EIN3 [11]. The protein kinase OST1 (open stomata 1) was recently found to phosphorylate ICE1 under cold stress and to thereby stabilize and activate ICE1 activity [12]. SIZ1 (SAP and Miz 1), a SUMO (small ubiquitin related modifier) E3 ligase, can stabilize ICE1 through sumoylation [13]. ICE1 protein stability and activity can also be regulated by E3 ligase HOS1 (high expression of osmotically responsive gene1)-mediated protein ubiquitination and proteosomal degradation under cold stress [14].

RNA-seq technology is increasingly being used to characterize transcriptomic events in plants. RNA-seq can provide transcriptomic information in the absence of a reference genome, and thus it is particularly useful in non-model species, whose genomic sequences are often unavailable. For many crops and other economically important plants, a period of unexpected low temperature may cause damage to plants and result in great losses to farmers. Recently, a number of studies have characterized cold responsive transcriptomes of these plants, including important crops such as *Prunus dulcis Mill.* (Almond) [15]*, Beta vulgaris L.* (Sugar beet) [16] and *Poncirus trifoliata* (L.) Raf. (Trifoliate orange) [17] and other plants with high economic value, such as *Eucalyptus dunnii* [18], *Chrysanthemum nankingense* [19], and *Lilium longiflorum* (Easter lily) [20, 21]. These studies suggested that the gene expression responses of plants to cold are complex and involve numerous cellular processes, such as carbohydrate metabolism, protein metabolism, calcium signaling and hormonal changes [17–20].

In this study, we performed *de novo* transcriptome assembly of *O. americanum* var*. pilosum* and compared its transcriptomes under normal and chilling conditions to investigate how *O. americanum* var*. pilosum* responds to low temperature stress. In the *de novo* assembled transcriptome of *O. americanum* var*. pilosum*, we identified 42,816 active transcribed unigenes and found that the expression of 5179 unigenes was up- or down-regulated in response to low temperature. To understand the potential involvement of the CBF pathway in the cold response of *O. americanum* var*. pilosum*, we reconstructed the CBF pathway by using our *de novo* assembled transcriptome and compared the expression of CBF pathway factors before and after chilling treatment. We found that none of the identified COR gene orthologs was induced by cold in *O. americanum* var*. pilosum*, which is consistent with the cold sensitive phenotype of this plant*.* In contrast, many of the cold regulated genes have functions related to protein translation and cellular metabolism, suggesting that cold temperature affects this chilling sensitive plant by altering protein synthesis and metabolism.

## Results and discussion

### Response of *O. americanum* var*. pilosum* to chilling stress

**Fig. 1:**
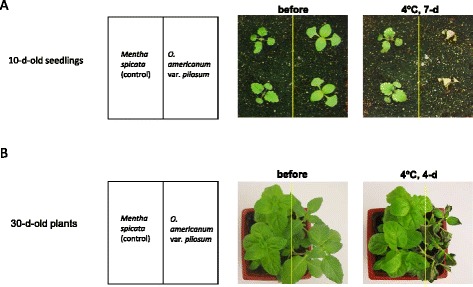
Chilling sensitivity of (**a**) 10- and (**b**) 30-day-old *O. americanum* var*. pilosum* plants. *Mentha spicata* was used as the control

Seeds of *O. americanum* var*. pilosum* were collected from Funan County of Anhui Province, China. Seeds were germinated on MS agar medium. Then 5-day-old seedlings were transferred to soil and grown in a growth room with a 16 h light/8 h dark photoperiod at 22 °C. Chilling treatment was performed in a climate chamber with a 16 h light/8 h dark photoperiod at 4 °C. During chilling treatment, control plants were kept at 22 °C. Mint (*Mentha spicata*, family Lamiaceae) belonging to the same family with *O. americanum* var*. pilosum* was used as a control (a chilling-tolerant plant) in the chilling sensitivity test*.* As shown in Fig. 1a, 10-day-old *O. americanum* var*. pilosum* and control plants appeared healthy before they were subjected to chilling stress. After a chilling stress of 4 °C for 7 d, the control seedlings but not the *O. americanum* var*. pilosum* seedlings survived, indicating that *O. americanum* var*. pilosum* is highly susceptible to chilling jury. When 30-day-old plants were subjected to chilling stress, the results once again indicated that chilling sensitivity is greater for *O. americanum* var*. pilosum* than for the control (Fig. 1b).

### RNA sequencing and *de novo* transcriptome assembly

Total RNA was isolated from both untreated (control) and chilled (4 °C, 12 h) 30-day-old *O. americanum* var*. pilosum* leaves. RNA sequencing with Illumina HiSeq2500 was performed for two biological replicates of control and chilled samples, which were designated control_rep1, control_rep2, chilling_rep1, and chilling_rep2. About 40 million paired-end reads were produced for each RNA-seq sample (Table 1). Clean reads were acquired from initial paired-end reads after low quality regions (Q < 20), PCR duplicates, and adaptor sequences were trimmed. For each control sample, approximately 30 million clean reads containing a total of 3 billion nucleotides were obtained (Table 1). For each chilling-treated sample, approximately 27 million clean reads containing a total of 2.7 billion nucleotides were obtained (Table 1). Biological replicates produced comparable data (Table 1).

**Table 1:** Sequencing output statistics in four *O. americanum* var*. pilosum* leaf samples from plants that were not chilled (Control_rep1 and 2) or chilled (Chilling _rep 1 and 2)

	Sample			
Variable	Control_rep1	Control_rep2	Chilling_rep1	Chilling_rep2
Number of raw reads	40,417,224	41,271,492	39,558,950	40,285,850
Number of clean reads	30,413,692	30,946,028	26,653,114	27,010,008
Percentage of reads kept	75	75	67	67
Size of clean reads (bp)	3,071,782,892	3,125,548,828	2,691,964,514	2,728,010,808
Number of total clean reads	115,022,842			

We pooled all high quality reads (115,022,842) from the four samples to perform the *de novo* transcriptome assembly (Table 1). With the Trinity program [22], 93,850 transcripts were assembled with an N50 length of 1283 bp, an average transcript length of 958 bp, and a maximal transcript length of 9,543 bp (Table 2). We chose the longest isoform of each gene to construct the unigene set. After isoforms were considered, these assembled transcripts were predicted to be produced from a total of 42,816 unigenes (Additional file 1: Table S1). The mean size of the unigenes was approximate 947 bp, and their lengths ranged from 300 bp to 9,543 bp (Table 2). We compared transcriptome of *O. americanum* var*. pilosum* with that of another species of *Ocimum, O. sanctum*. Previous published transcriptome of *O. sanctum* was used in this analysis [5, 6]. We found that 71 % of unigenes (30577) in *O. americanum* var*. pilosum* got hits (*E-value* < =1.0E-05) in the transcriptome of *O. sanctum*, suggesting high similarity between these two species*.* The 29 % of unigenes that don’t get hits may reflect the genomic diversity between the two different species.

**Table 2:** Statistics of transcriptome assembly and predicted unigenes

Variable	Total number of assembled transcripts	Number of predicted unigenes
Number	93,850	42,816
Size of data (bp)	89,919,719	40,526,158
Minimum length (bp)	201	300
Maximum length (bp)	9543	9543
Mean length (bp)	958	947
N50 length (bp)	1283	1346

**Fig. 2:**
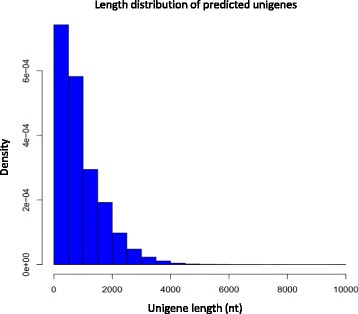
The distribution of unigene lengths for *O. americanum* var*. pilosum*

As indicated by the length distribution of *O. americanum* var*. pilosum* unigenes (Fig. 2), most unigenes (38783, 90.6 %) had < 2000 nucleotides, and the number of unigenes decreased as gene length increased. Genes with only one transcript were considered to be distinct singletons, while genes with multiple transcripts were considered to be distinct clusters. Among 42,816 unigenes, 24,196 unigenes were distinct singletons, and about 18,620 unigenes were distinct clusters.

### Annotation and classification of *O. americanum* var*. pilosum* unigenes

**Fig. 3:**
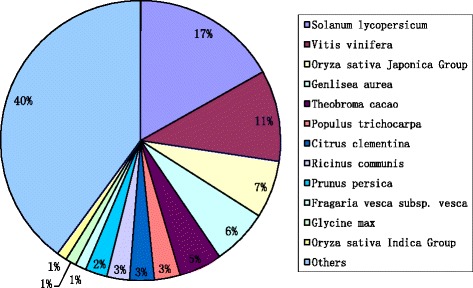
Similarity of *O. americanum* var*. pilosum* sequences with those of other species

To identify the putative functions of *O. americanum* var*. pilosum* unigenes, we carried out functional annotation by using a BLASTx search (E-value < =1.0E-05) against several protein databases: NCBI non-redundant (nr) database, Swiss-Prot database, Gene Ontology (GO), Kyoto Encyclopedia of Genes, Genomes (KEGG) database, and Cluster of Orthologous Group (COG) database. Of the 42,816 unigenes, 71.5 % (30,628 unigenes) were successfully aligned to known proteins in the nr database (Table 3). In the other four databases (SwissProt, GO, KEGG, and COG), 24,531, 10,172, 8,533, and 28,853 unigenes were successfully aligned to known proteins (Table 3), respectively. Overall, 30,748 (71.8 %) unigenes were significantly similar to known proteins in the five databases, while 12,068 (28.19 %) unigenes were not similar to any known protein in the five databases. Among the five databases, the nr database produced the largest number of annotations; of the 30,748 annotated unigenes, 30628 (99.6 %) were annotated in the nr database. In comparison with other species, *O. americanum* var*. pilosum* unigenes showed the highest similarity with sequences from *Solanum lycopersicum* (7211) and *Vitis vinifera* (4514), but also were similar to sequences from other species (Fig. 3 and Additional file 2: Table S2).

**Table 3:** Annotation statistics of *O. americanum* var. *pilosum* unigenes

Database	Unigene number	Number of DEGs
Nr	30,628	4457
Swisprot	24,531	3700
KEGG	8533	1399
Gene Ontology	10,172	1450
COG	28,853	4261
Total	30,748	4465

**Fig. 4:**
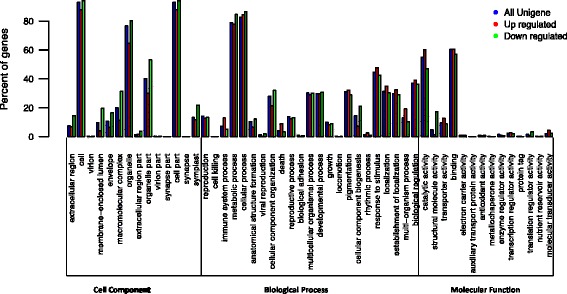
Histogram of GO (gene ontology) classifications of assembled unigenes and up- and down-regulated unigenes of *O. americanum* var*. pilosum* in response to low temperature

We performed a GO analysis to classify the functions of the *O. americanum* var*. pilosum* unigenes. A total of 10172 unigenes were successfully annotated with GO terms and were classified into three major GO categories: Biological Processes (BP), Cell Component (CC), and Molecular Function (MF). They were further assigned to 51 subcategories based on GO level 2. The dominant subcategories for the classified genes were ‘cell’ (93.1 %) and ‘cell part’ (93.1 %) for the CC category; ‘cellular process’ (82.6 %), ‘metabolic process’ (78.8 %), and ‘response to stimulus’ (44.7 %) for the BP category; and ‘binding’ (60.4 %), ‘catalytic activity’ (54.9 %), and ‘transporter activity’ (9.5 %) for the MF category (Fig. 4). To identify active biochemical pathways in *O. americanum* var*. pilosum*, KEGG analysis was carried out; KEGG-annotated unigenes (8533) were classified to 273 pathways (>10 associated unigenes) (Additional file 3: Table S3). Among these pathways, the five most represented were “Carbon metabolism”, “Biosynthesis of amino acids”, “Ribosome”, “Protein processing in endoplasmic reticulum”, and “Plant-pathogen interaction” (Additional file 4: Figure S1).

In summary, all these annotation and classification analyses can provide valuable information to further investigate gene functions, metabolic processes, and active pathways of *O. americanum* var*. pilosum*.

### Differentially expressed genes in chilling-treated *O. americanum* var*. pilosum* plants

Using our *de novo* assembled transcriptome as a reference, we identified putative genes expressed in control and chilling-treated plants. In control_rep1 and control_rep2 samples, 40,372 and 40,407 expressed putative genes (FPKM > =1) were detected, and 39334 were expressed in both samples. In chilling_rep1 and chilling_rep2 samples, 40,004 and 40,043 expressed putative genes (FPKM > =1) were detected, and 39,028 were expressed in both samples. The high similarity between the two biological replicates for either control or chilling-treated samples indicated that the RNA-seq results were consistent. The consistency was also supported by FPKM (fragments per kilobase of gene per million mapped reads) correlation analysis between the two biological replicates (r > 0.99) (Additional file 5: Figure S2).

To begin to explore the molecular mechanisms of cold stress response of *O. americanum* var*. pilosum*, we identified genes that were differentially expressed in seedlings grown under normal vs. chilling temperatures. Compared with the control, 5,179 differentially expressed genes (DEGs) with at least 2-fold changes (false discovery rate [FDR] < 0.01) were identified in chilling-treated samples with the edgeR package. Of these DEGs, 2,344 were up-regulated and 2,835 were down-regulated in chilling-treated plants. Functional annotation with five databases was also executed on these DEGs, and about 86 % of them (4,465 DEGs) were successfully annotated (Table 3 and Additional file 1: Table S1).

About 608 up-regulated DEGs and 842 down-regulated DEGs were successfully annotated with GO (Table 3 and Additional file 1: Table S1). Their GO level 2 distributions are shown in Fig. 4. Up-regulated and down-regulated DEGs had a similar distribution pattern, which was also similar to that of all GO annotated unigenes (Fig. 4).

**Fig. 5:**
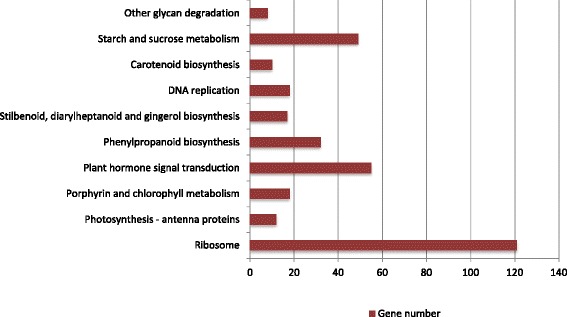
KEGG pathway classification of differentially expressed unigenes of *O. americanum* var*. pilosum*

To better understand the biological function and correlation of these DEGs, we conducted an enrichment analysis with the KEGG pathway, which assigned the DEGs to 10 pathways (Fig. 5). Among these pathways, “Ribosome”, “Plant hormone signal transduction”, “Starch and sucrose metabolism”, and “Phenylpropanoid biosynthesis” were the most highly represented (Table 4). The “Ribosome” pathway (ko03010) had the largest number of DEGs, suggesting that translation in *O. americanum* var*. pilosum* is substantially influenced by low temperature. The pathway with the second largest number of DEGs was “Plant hormone signal transduction” (ko04075), indicating that plant hormones in *O. americanum* var*. pilosum* play important roles in the response to chilling stress. Sucrose and starch metabolism (ko00500) was the pathway with the third largest number of DEGs; this was not surprising because chilling affects carbon assimilation and sucrose contributes to chilling tolerance by stabilizing plasma membranes [23]. *Ocimum* spp. are commonly used as spices and as sources of essential oils because these plants synthesize large quantities of phenylpropanoid compounds [24]. Interestingly, the pathway with the fourth largest number of DEGs was “Phenylpropanoid biosynthesis” (ko00940), suggesting that the composition of aromatic compounds may change under cold stress.

**Table 4:** KEGG pathway enrichment of DEGs

Pathway ID	Description	*P*-value	Gene number
ko03010	Ribosome	2.30E-25	121
ko04075	Plant hormone signal transduction	6.31E-04	55
ko00500	Starch and sucrose metabolism	4.88E-03	49
ko00940	Phenylpropanoid biosynthesis	7.59E-04	32
ko00860	Porphyrin and chlorophyll metabolism	4.01E-04	18
ko03030	DNA replication	3.25E-03	18
ko00945	Stilbenoid, diarylheptanoid and gingerol biosynthesis	2.08E-03	17
ko00196	Photosynthesis - antenna proteins	3.66E-08	12
ko00906	Carotenoid biosynthesis	3.86E-03	10
ko00511	Other glycan degradation	8.91E-03	8

In general, analyses towards DEGs in response to cold will help to understand how gene expression in *O. americanum* var*. pilosum* is influenced by chilling treatment.

### Validation of DEGs in chilling-treated *O. americanum* var*. pilosum* plants

**Fig. 6:**
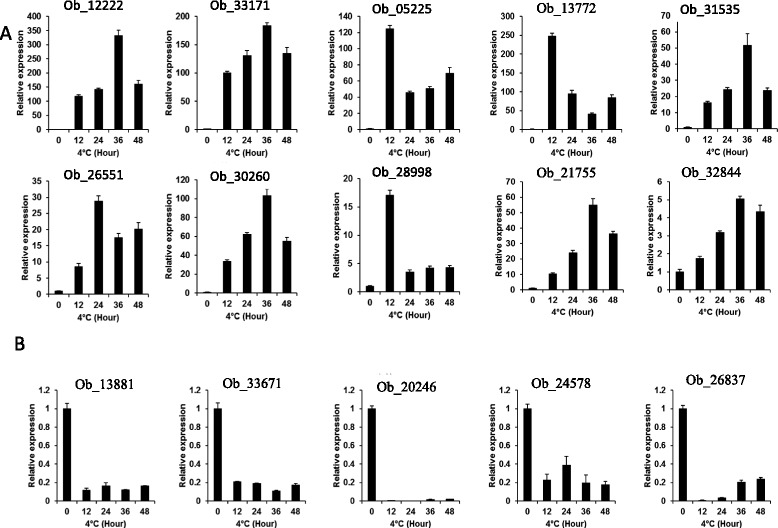
The expression of *O. americanum* var*. pilosum* genes in response to chilling at 4 °C for 0 to 48 h as determined by qRT-PCR. **a** Expression of the 10 most up-regulated genes. **b** Expression of the five most down-regulated genes

We checked the functional annotations of those DEGs that exhibited the greatest difference in expression in response to chilling treatment. The 10 most up- and down-regulated genes and their annotations are listed in Table 5. Ob_12222, a putative ortholog of PID-binding protein 1 (*PBP1*) in *Arabidopsis,* was the gene that was most induced by chilling treatment. This indicates an interplay between auxin signaling and cold response in *O. americanum* var*. pilosum* because PBP1 is related to auxin signaling. More specifically, PBPI can stimulate the autophosphorylation activity of PID (PINOID) protein serine/threonine kinase, which is a key component in auxin signaling [25], and auxin plays an important role in cold stress inhibition of plant growth and development [26]. To validate our RNA sequencing results, we performed individual qRT-PCR to examine the expression of the 10 most up-regulated genes and the five most down-regulated genes after chilling for 0, 12, 24, 36, and 48 h. All 10 up-regulated genes were well induced by chilling, although their expression changed with the length of the chilling treatment (Fig. 6a). Consistent with the RNA-seq results, all five of the most down-regulated genes were repressed by chilling (Fig. 6b). The consistency between qRT-PCR results and RNA-seq analyses helps confirm the validity of our de novo assembled transcriptome and our analysis of cold stress regulation of the transcriptome.

**Table 5:** The 10 most up- and down-regulated *O. americanum* var. *pilosum* genes after cold treatment

Unigene ID	Type	Log2 (fold-change)	Name	Description
Ob_12222	up	8.01	PBP1	Calcium-binding protein PBP1
Ob_33171	up	6.82	At2g40140	Zinc finger CCCH domain-containing protein 29
Ob_05225	up	6.54	NA	NA
Ob_13772	up	6.41	LIR1	Light-regulated protein
Ob_31535	up	6.32	NA	Brassinosteroid-regulated protein BRU1
Ob_26551	up	6.23	WRKY53	Probable WRKY transcription factor 53
Ob_30260	up	5.53	MYC4	Transcription factor MYC4
Ob_28998	up	5.36	BBD2	Bifunctional nuclease 2
Ob_21755	up	5.36	NA	NA
Ob_32844	up	5.36	RVE1	Protein REVEILLE 1
Ob_13881	down	−6.97	NA	NA
Ob_33671	down	−6.50	CYP71A8	Cytochrome P450 71A8
Ob_20246	down	−5.72	CAB36	Chlorophyll a-b binding protein 36, chloroplastic
Ob_24578	down	−5.59	CDF3	Cyclic dof factor 3
Ob_26837	down	−5.47	COL5	Zinc finger protein CONSTANS-LIKE 5
Ob_36212	down	−5.43	WAXY	Granule-bound starch synthase 1, chloroplastic/amyloplastic
Ob_38127	down	−5.27	NA	Retrovirus-related Pol polyprotein from transposon TNT 1-94
Ob_34404	down	−5.15	DSPP	Dentin sialophosphoprotein
Ob_20836	down	−4.97	SAT1	Serine acetyltransferase 1, chloroplastic
Ob_27478	down	−4.96	UGT83A1	UDP-glycosyltransferase 83A1

### *O. americanum* var*. pilosum* unigenes involved in the CBF cold response pathway

The CBF transcriptional cascade is an important pathway for the regulation of gene expression under low temperature in diverse plant species. Using our RNA-seq data for *O. americanum* var*. pilosum* and Blastx, we identified CBF-pathway genes with the following parameters: an expectation value of 1e-5, an identity value > 30 %, a coverage value > 80 %, and a protein length difference < 2x. A total of 39 unigenes were identified as putative orthologs of genes in the CBF pathway, and their corresponding CBF-pathway orthologs are listed in Table 6. In *O. americanum* var*. pilosum*, Ob_28165 and Ob_11817 were found to be similar to ICE1, which can be modified by phosphorylation and sumolation when plants are exposed to low temperature. The enzyme responsible for ICE1 sumoylation, SIZ1, has four similar unigenes: Ob_17220, Ob_17219, Ob_35232, and Ob_03308. The modified ICE1 can activate *CBF* transcription and repress the *CBF* transcription inhibitor, MYB15. In our assembled transcriptome, 10 unigenes (Ob_36284, Ob_07479, Ob_16876, Ob_32681, Ob_06567, Ob_08721, Ob_09654, Ob_08732, Ob_15732, and Ob_38631) with high similarity to the MYB15 sequence were identified, and six unigenes (Ob_05376, Ob_29030, Ob_04623, Ob_07111, Ob_16900, and Ob_17034) were matched to known *CBF* gene sequences (Table 6). The degradation of ICE1 can be facilitated by ubiquitination by HOS1, and thus HOS1 negatively regulates *CBF* expression. One unigene, Ob_33354, had high similarity with HOS1. CBFs can regulate the cold induction of ZAT10, a transcription factor involved in regulating the expression of a subset of cold-responsive genes (8). Two putative *ZAT10* orthologs were found in *O. americanum* var*. pilosum*: Ob_13389 and Ob_17132. Sequences of the following eight expressed unigenes were similar to those of known COR genes, including *COR15A*, *COR47*, and *KIN1*: Ob_16474, Ob_09195, Ob_10639, Ob_11797, Ob_11798, Ob_29046, Ob_39125, and Ob_29616.

**Table 6:** Unigenes matched to known CBF-pathway factors

Unigene	Ortholog	Identity (%)	E-value	Control_rep1	Control_rep2	Chilling_rep1	Chilling_rep2	Species
Ob_28165	ICE1	32.39	7E-23	3.99	2.77	1.69	1.5	*Camellia sinensis*
Ob_11817	ICE1	43.3	9E-42	3.44	3.82	0.47	0.23	*Ageratina adenophora*
Ob_17220	SIZ1	71.95	8E-87	3.35	3.29	4.19	5.16	*Medicago truncatula*
Ob_17219	SIZ1	69.74	6E-95	10.61	11.98	12.85	12.44	*Medicago truncatula*
Ob_35232	SIZ1	46.97	0	20.28	19.72	26.04	25.37	*Triticum urartu*
Ob_03308	SIZ1	41.07	1E-31	1.03	1.77	1.05	1.03	*Medicago truncatula*
Ob_36284	MYB15	37.16	7E-27	19.67	19.83	24.17	22.48	*Oryza sativa*
Ob_07479	MYB15	45.26	4E-23	0	0	6.01	5.56	*Oryza sativa*
Ob_16876	MYB15	83.81	5E-67	1.67	1.31	2.03	1.33	*Oryza sativa*
Ob_32681	MYB15	52.71	7E-41	14.13	14.66	19.59	19.57	*Oryza sativa*
Ob_06567	MYB15	54.26	1E-45	0.67	0.66	0.68	0.34	*Oryza sativa*
Ob_08721	MYB15	42.11	3E-32	0.26	0.26	3.97	4.7	*Oryza sativa*
Ob_09654	MYB15	51.72	1E-37	0	0	0.92	0.9	*Oryza sativa*
Ob_08732	MYB15	56.76	2E-42	2.21	1.45	3.37	3.32	*Oryza sativa*
Ob_15732	MYB15	59.43	1E-44	1.33	1.31	1.01	1	*Oryza sativa*
Ob_38631	MYB15	66.3	5E-42	1.24	0	0.42	1.65	*Oryza sativa*
Ob_33354	HOS1	58.51	4E-87	54.04	52.4	61.79	59.4	*Medicago truncatula*
Ob_05376	CBF1	33.33	5E-11	0	0	0.38	0.37	*Arabidopsis thaliana*
Ob_29030	CBF1	34.02	2E-11	42.71	39.55	211.72	218.64	*Solanum lycopersicum*
Ob_04623	CBF2	52.38	3E-29	1.12	1.84	0.38	0.75	*Solanum lycopersicum*
Ob_07111	CBF2	75	9E-54	0	0	0.72	1.42	*Solanum lycopersicum*
Ob_16900	CBF3	36.78	4E-08	1.66	2.86	0	0	*Hordeum vulgare* *subsp. vulgare*
Ob_17034	CBF3	38.85	1E-14	19.73	18.92	7.1	5.45	*Hordeum vulgare* *subsp. vulgare*
Ob_13389	ZAT10	54.24	2E-33	0	0.31	6.79	10.51	*Arabidopsis thaliana*
Ob_17132	ZAT10	35.11	3E-12	1.09	0.59	7.01	6.79	*Cicer arietinum*
Ob_16474	COR15A	39.51	5E-6	7.71	7.45	2.73	2.69	*Arabidopsis thaliana*
Ob_09195	COR47	41.67	1E-09	8.34	6.96	0.38	0.62	*Arabidopsis thaliana*
Ob_10639	COR47	40.41	5E-27	9.95	11.87	1.63	1.78	*Arabidopsis thaliana*
Ob_11797	COR47	41.27	8E-18	2.07	1.69	4.55	4.48	*Arabidopsis thaliana*
Ob_11798	COR47	46.07	3E-14	1.12	0.74	1.9	3.75	*Arabidopsis thaliana*
Ob_29046	COR47	40.09	8E-32	177.38	175.3	195.61	192.62	*Arabidopsis thaliana*
Ob_39125	COR47	42.64	3E-18	6.42	7.32	8.61	9	*Arabidopsis thaliana*
Ob_29616	KIN1	37.88	6E-11	1.85	2.43	4.39	5.87	*Arabidopsis thaliana*

**Fig. 7:**
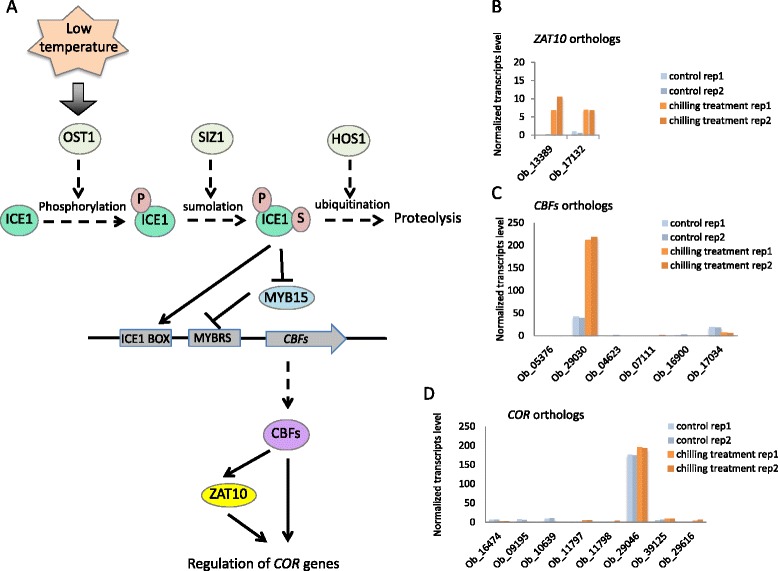
Reconstruction of the CBF pathway in *O. americanum* var*. pilosum* and the relative expression of 16 unigenes putatively involved in the CBF signaling pathway in response to control and chilling treatment. **a** Diagram showing CBF pathway. **b**–**d** Relative expression of *ZAT10*, *CBF*, and *COR* orthologues in control and chilling treatment. For **b**–**d**, the results of two biological replicates are shown

For unigenes with similarity to CBF-pathway factors, normalized transcript levels in control and chilling-treated samples are shown in Fig. 7. When >2-fold change was used as a cutoff, two putative *ICE1* orthologs (Ob_28165 and Ob_11817) and one putative *CBF3* ortholog (Ob_17034) were slightly down-regulated, while one putative *CBF1* ortholog (Ob_29030) and two putative *ZAT10* orthologs (Ob_13389 and Ob_17132) were up-regulated after chilling treatment. Ob_29030 (similar to *CBF1*) was dramatically induced by chilling treatment (Additional file 7: Figure S3. and Fig. 7), while transcript levels of all the other putative *CBF* orthologs were very low. CBF can activate *ZAT10* (8). Consistent with the induced expression of the putative CBF ortholog (Ob_29030), the expression of two putative *ZAT10* orthologs (Ob_13389 and Ob_17132) was increased in response to chilling treatment. The transcript levels of the two putative ICE1 orthologs were reduced to different degrees after chilling treatment, although they both were expressed at low levels in control samples. Among the 10 putative *MYB15* orthologs, transcript levels for two (Ob_07479 and Ob_08721) were slightly increased by chilling treatment (Additional file 7: Figure S3), and this increase might be explained by the decreased expression of its negative regulators, putative ICE1 orthologs. One putative *CBF3* ortholog (Ob_17034), as a possible target of putative ICE1 and MYB15 orthologs, was slightly repressed by chilling treatment. Transcript levels of putative SIZ1 and HOS1 orthologs did not change in response to chilling treatment (Additional file 7: Figure S3). Interestingly, none of the identified COR orthologs in *O. americanum* var*. pilosum* was substantially induced by chilling treatment (Fig.7d).

## Conclusions

*O. americanum* var*. pilosum* is an aromatic plant and a popular vegetable in central and southern parts of China. It is very sensitive to low temperatures. Here, we performed *de novo* transcriptome assembly of *O. americanum* var*. pilosum* using the Trinity program and obtained 42,816 assembled unigenes. By analyzing the genome-wide transcriptome under low temperature, we identified several thousand potential cold-responsive unigenes and 10 pathways containing DEGs under chilling treatment. Our analysis of the DEGs suggested that cold temperature significantly affects protein translation and cellular metabolism in this chilling sensitive species. Although most genes involved in the ICE1-CBF-COR pathway have orthologs in *O. americanum* var*. pilosum*, none of the identified orthologs of COR genes was induced by low temperature, which is consistent with the lack of cold tolerance in this plant. In summary, we have profiled the high-resolution expression pattern of *O. americanum* var*. pilosum* under normal and chilling conditions. Our results should be useful for future research concerning the molecular mechanisms of low temperature responses in *O. americanum* var*. pilosum.*

## Methods

### Plant materials

Seeds of *O. americanum var. pilosum* in this study were collected from Funan County of Anhui Province, China. Seeds germinated on MS agar medium. Then 5-day old seedlings were transferred to soil and grown in a growth room with a 16-h-light/8-h-dark photoperiod at 22 °C. Chilling treatment were performed in climate chamber with a 16-h-light/8-h-dark photoperiod at 4 °C. During chilling treatment, control plants were kept in the same conditions but at normal temperature (22 °C). RNA from 30-day-old plants was used in RNA-seq. For RT-PCR, young leaves of 6 individual one-month-old plants were collected at every indicated time points and immediately frozen in liquid nitrogen before RNA extraction. Plants used for RT-PCR and RNA-seq were harvested in two independent experiments. Biological replicates in RNA-seq were harvested at the same time.

### RNA sequencing and *de novo* transcriptome assembly

Total RNA was extracted from leaves of control and chilling (4 °C for 12 h) treated 30-day-old plants with the RNApure High-purity Total RNA Rapid Extraction Kit (Bioteke). Each treatment (± chilling) was represented by two biological replicates. Residual genomic DNA was removed from the total RNA with the Turbo DNA-Free kit (Ambion) following the manufacturer's instructions. The sequencing library construction and sequencing were performed in the Genomics Core Facilities of the Shanghai Center for Plant Stress Biology, SIBS, CAS (Shanghai, China) with Illumina HiSeq2500. Clean reads were acquired from initial paired-end reads after low quality regions (Q <20), PCR duplicates, and adaptor sequences were trimmed.

Inchworm, Chrysalis, and Butterfly modules of Trinity software [15, 20] were used for *de novo* transcriptome assembly. We first combined the sequencing reads from the four samples and applied Inchworm to assemble the RNA-seq data into contigs (unique sequences of transcripts) with the default K-mer parameter and minimum K-mers coverage of three. The resulting Inchworm contigs were bunched by Chrysalis into clusters; Chrysalis was then used to constructed complete de Bruijn graphs for each cluster. Finally, the final assembled transcripts for alternatively spliced isoforms were reconstructed by Butterfly by reconciling individual deBruijn graphs, and only transcripts ≥ 300 bp long were retained for further analysis.

### Annotation of *O. americanum* var*. pilosum* unigenes

Unigenes were first annotated by using BLASTX with an expectation value of 10^−5^ to search the following protein databases: NCBI nr protein database (NCBI non-redundant sequence database), SwissProt, and KEGG. Next, protein information and their functional annotations were retrieved for genes with the highest sequence similarity with *O. americanum* var*. pilosum* unigenes.

### Identification and annotation of DEGs

DEGs were identified based on the negative binomial distribution with the edgeR package [27]. We calculated the false discovery rate (FDR) values of genes firstly through edgeR, and mapped reads numbers of genes were used in this analysis. Genes with FDR ≤0.01 were considered as candidates. In addition, fragments per kilobase of gene per million mapped reads (FPKM) of these candidates was generated by using RSEM [28]. Finally, the fold change of FPKM was computed, and genes with the over or equal to 2-fold change were characterized as DEGs. Functional enrichment analyses were then performed on identified DEGs by using GOstats [29]. For Gene Ontology and KEGG pathway analysis, we used Hypergeometric test function (p value < 0.001) [30].

### qRT-PCR analysis of gene expression

With the RNApure High-purity Total RNA Rapid Extraction Kit (Bioteke), total RNA was extracted from leaves of 1-month-old plants treated at 4 °C for 0, 12, 24, 36, and 48 h. Residual genomic DNA was removed from the total RNA with the Turbo DNA-Free kit (Ambion) following the manufacturer's instructions. The first-strand cDNAs were synthesized from total RNA with the SupermoIII M-MuLV RT Kit (Bioteke) and were used as templates. A CFX96 Real-Time PCR Detection System (Bio-Rad) was used for real-time quantitative RT-PCR (qRT-PCR) with TransStart Tip Green qPCR SuperMix (TransGen Biotech) to confirm the identity of up- or down-regulated genes. Each experiment had three biological replicates, and the PCR conditions were as follows: 40 cycle of 95 °C for 3 min, 95 °C for 10 s, and 58 °C for 30 s. The DNA primers for probe amplification are listed in Additional file 6: Table S4. The comparative cycle threshold (ct) method was used to calculate gene expression levels, and the *TUBULIN* ortholog Ob_23367 was used as reference.

### Availability of supporting data

The data sets supporting the results of this article are available in the NCBI’s Gene Expression Omnibus (GSE68980).

## Additional files

Additional file 1: Table S1. List of annotated unigenes and lists of up-regulated genes and down-regulated genes in chilling-treated samples. (XLS 14564 kb). Additional file 2: Table S2. Similarities between *O. americanum *var*. pilosum *and other species. (XLS 70 kb). Additional file 3: Table S3. Pathways annotated by KEGG analysis. (XLS 221 kb). Additional file 4: Figure S1. The assignment of *O. americanum* var*. pilosum* unigenes to KEGG biochemical pathways. (PDF 114 kb) Additional file 5: Figure S2. Similarity analysis between two biological replicates of control and chilling-treated samples. (A) Correlation of the RNA-sequencing data between two biological replicates of control samples, between two biological replicates of chilling-treated samples, and between control samples and chilling-treated samples of *O. americanum* var*. pilosum*. (B) PCA analysis was performed to show similarity among different RNA-sequencing data. (PDF 104 kb) Additional file 6: Table S4. Primer information. (XLSX 38 kb). Additional file 7: Figure S3. The relative expression of 17 *O. americanum* var*. pilosum* unigenes involved in the CBF pathway in untreated plants (grey bars) and in chilling-treated plants (orange bars). Values for two biological replicates are shown. (PDF 176 kb)
