# Supplementary figures and images for: De novo assembly and analysis of the transcriptome of Ocimum americanum var. pilosum under cold stress
Source: BMC Genomics. 2016 Mar 9;17:209. doi: 10.1186/s12864-016-2507-7 (PMC4784345; doi:10.1186/s12864-016-2507-7)

Figure S1

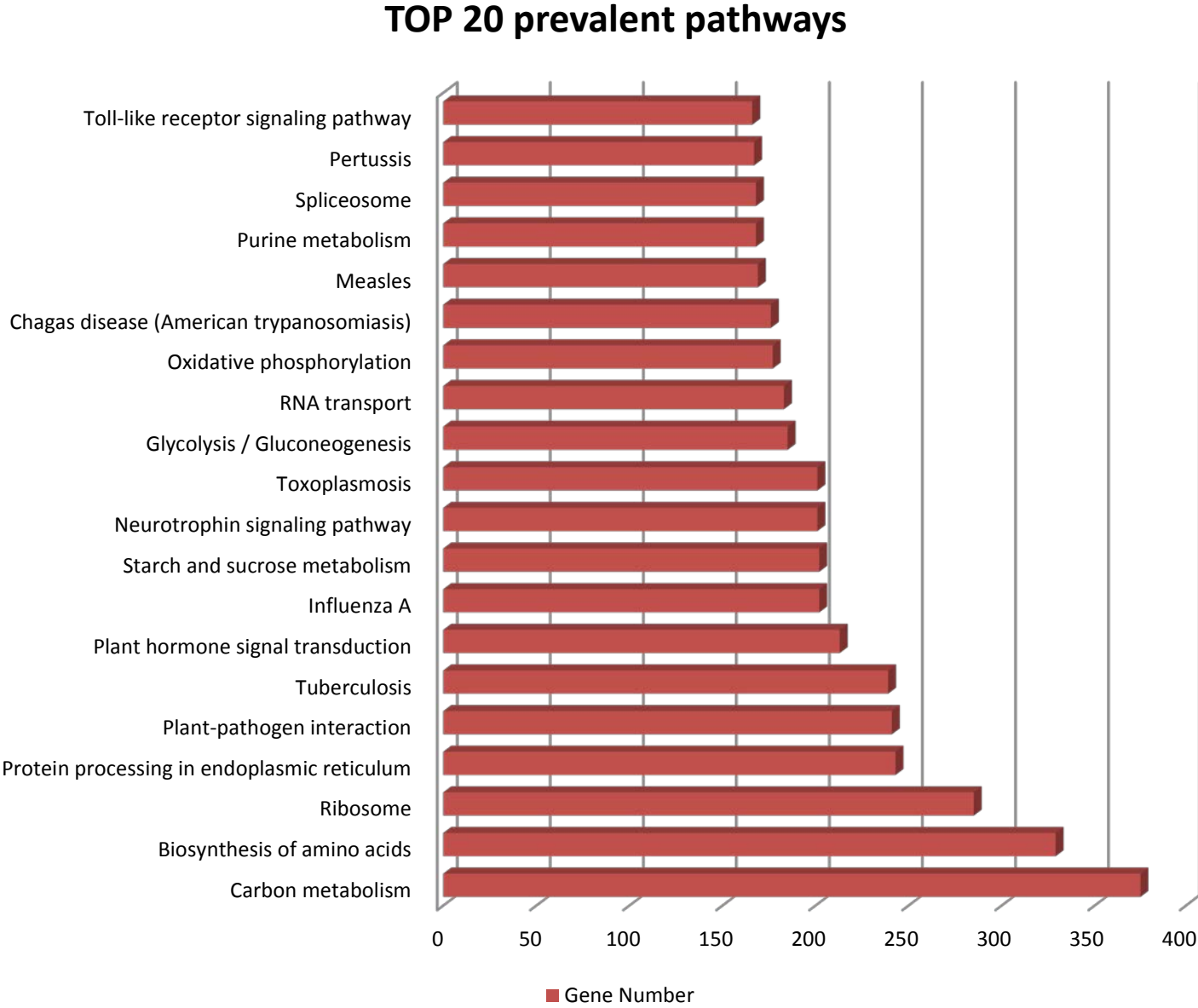

Supplement: Additional file 4: Figure S1. — The assignment of O. americanum var. pilosum unigenes to KEGG biochemical pathways. (PDF 114 kb) [file 12864_2016_2507_MOESM4_ESM.pdf]

Figure S2

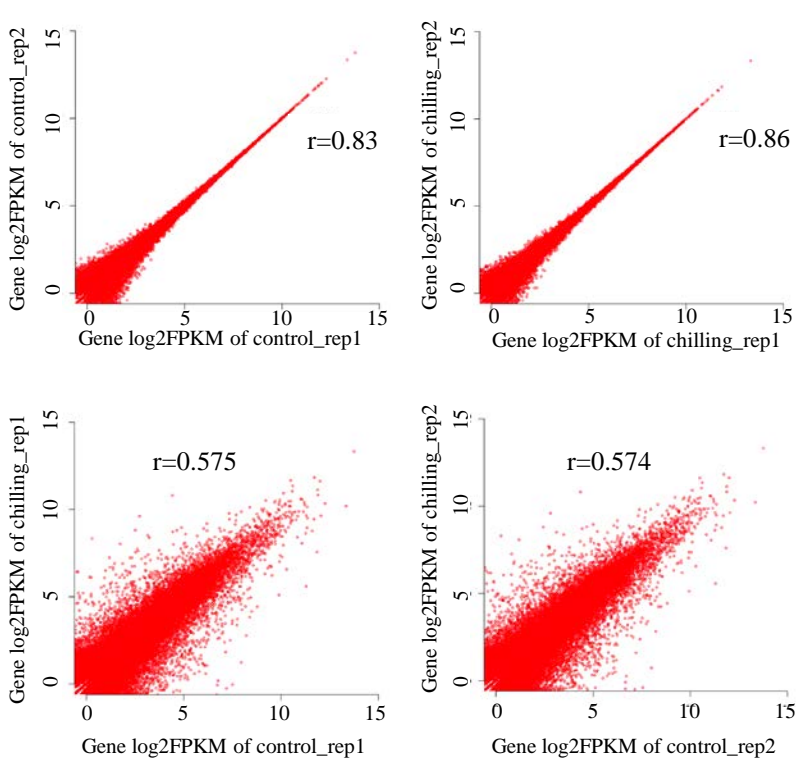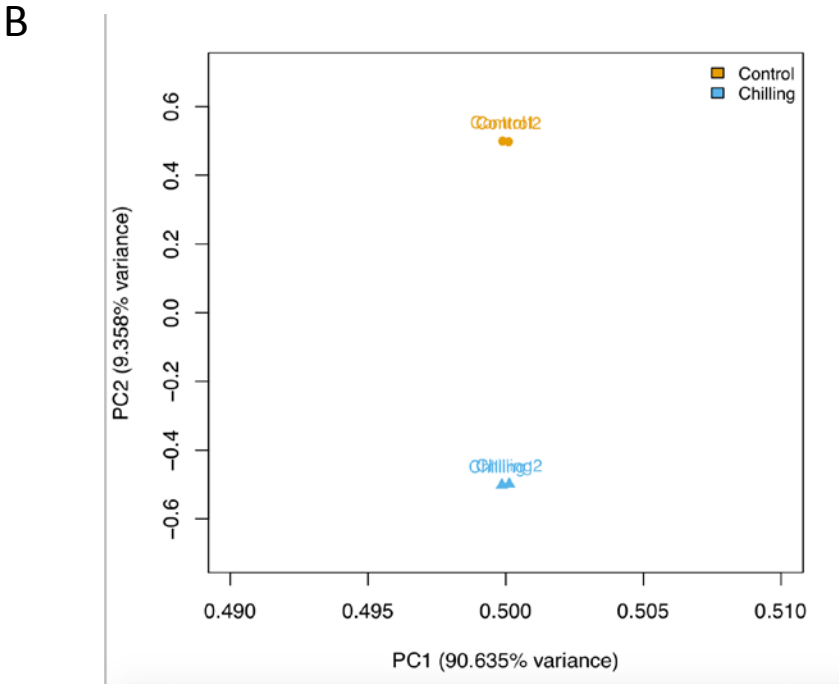

Supplement: Additional file 5: Figure S2. — Similarity analysis between two biological replicates of control and chilling-treated samples. (A) Correlation of the RNA-sequencing data between two biological replicates of control samples, between two biological replicates of chilling-treated samples, and between control samples and chilling-treated samples of O. americanum var. pilosum. (B) PCA analysis was performed to show similarity among different RNA-sequencing data. (PDF 104 kb) [file 12864_2016_2507_MOESM5_ESM.pdf]

Figure S3

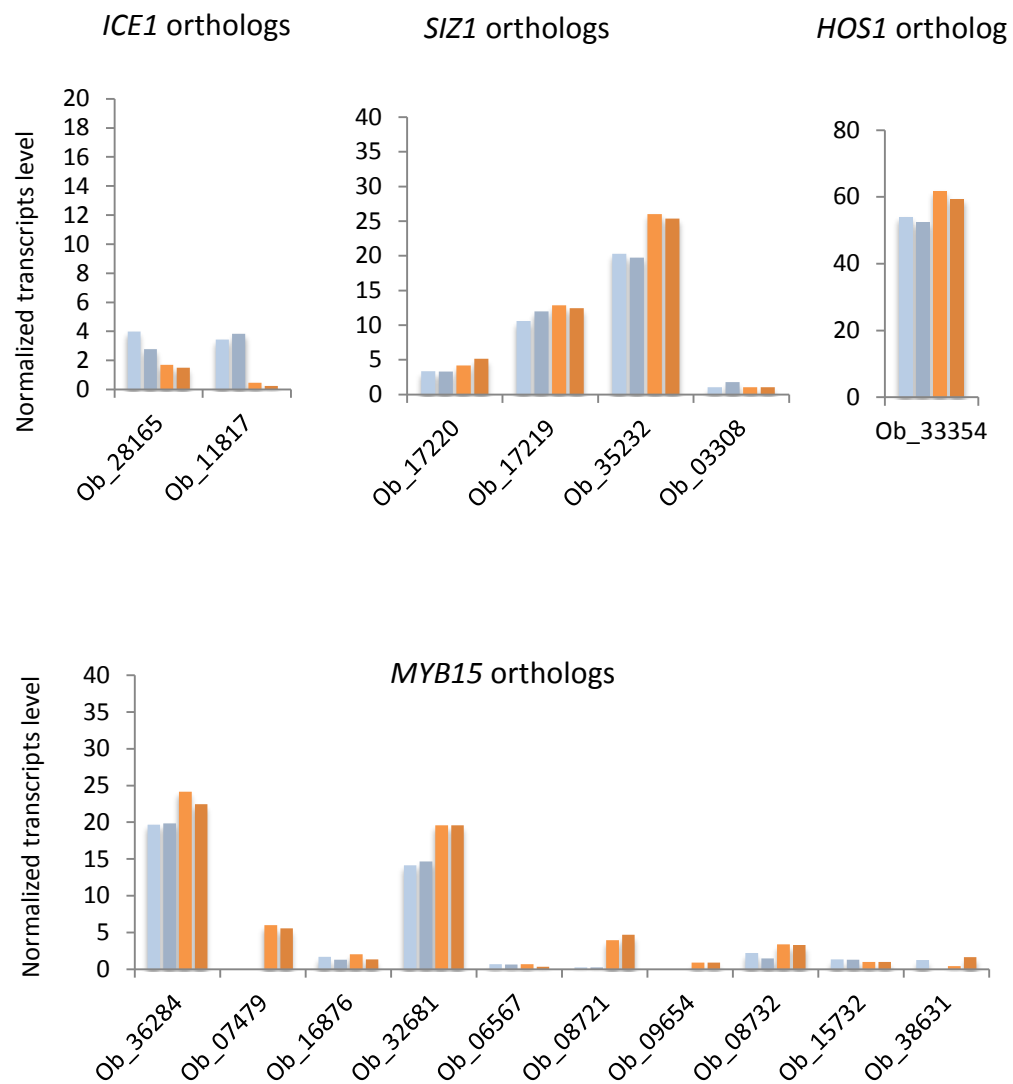

Supplement: Additional file 7: Figure S3. — The relative expression of 17 O. americanum var. pilosum unigenes involved in the CBF pathway in untreated plants (grey bars) and in chilling-treated plants (orange bars). Values for two biological replicates are shown. (PDF 176 kb) [file 12864_2016_2507_MOESM7_ESM.pdf]
